# Addressing the risk of a new wave: managing COVID-19 cases associated with XBB variants in china – a correspondence

**DOI:** 10.1097/MS9.0000000000000943

**Published:** 2023-06-08

**Authors:** Nicholas Aderinto, Gbolahan Olatunji

**Affiliations:** a Department of Medicine and Surgery, Ladoke Akintola University of Technology; b Department of Medicine and Surgery, University of Ilorin

*Dear Editor,*

The WHO has declared the end of the coronavirus disease 2019 (COVID-19) pandemic as a global health emergency. However, concerns have emerged due to the highly contagious XBB subvariant in China and internationally^1^. This subvariant is expected to cause a significant weekly surge of ~65 million infections in China^2^. Although China has ended its zero COVID policy, there are fears of its possible reinstatement and the implications of the XBB subvariant.

The XBB.1.5 subvariant has gained attention in China due to its increased transmissibility. Among the 199 identified cases of XBB from October to December, four were attributed to XBB.1.5, but no localised infections were detected. Surveillance data from China’s disease control authorities indicate that despite the rising prevalence of XBB variants, there has not been a substantial increase in new cases, hospitalisations, or fatalities^3^. Neutralising antibodies from previous viral strains remain effective against XBB and other variants for around three months.

From 1 December to 20 April, China reported 32 993 domestic cases with available genomic sequencing data. Weekly reports identified all Omicron variations, including 117 subvariants. The dominant strains were BA.5.2 and BF.7, and meticulous analysis found 603 variant cases, including 12 newly identified subvariants. XBB.1.16, known as ‘Arcturus’, was initially detected in India and is spreading in China and other regions^4^. China discovered 15 cases of Arcturus in six days in April. Although Arcturus has been found in samples from Hong Kong, authorities assure that there has been no increase in severity, but it has been associated with conjunctivitis in children.

The rapid transmission of this variant poses a significant public health threat, disrupting organisations, education, and daily activities and potentially causing economic instability and social unrest^4^. Concerns arise about the effectiveness of existing vaccines against the variant, casting doubt on ongoing vaccination efforts. If the variant evades vaccine-induced immunity, it could undermine progress in controlling the spread and mitigating the impact of the virus.

Reinforcing vigilance and implementing measures like widespread testing, contact tracing targeted vaccination campaigns, and public health guidelines are crucial to control the spread, mitigate risks, and minimise the consequences of a resurgence in cases, particularly if reinfections occur despite prior exposure or vaccination.

## Understanding XBB variants

BJ.1 and BM.1.1.1 were recombined to generate XBB, with XBB.1.16 classified as an Omicron subvariant under surveillance^4^. Although the global prevalence of XBB.1.16 is gradually increasing, the overall risk assessment remains low. More research is needed to understand its growth advantage, neutralisation capacity, and potential changes in disease severity. XBB exhibits higher fitness and immunological tolerance to breakthrough infections than its parental lineages. The XBB sublineage includes XBB.1.5, which raises concerns due to its recent identification and potential antibody evasion.

Monitoring and studying coronavirus variants are crucial to outbreak prevention and pandemic management. Understanding their evolutionary patterns and implications for public health allows personalised therapies and evaluation of their efficacy. Variant monitoring is essential for assessing the effectiveness of preventive interventions like vaccines and public health measures. Guide vaccine development and helps determine if adjustments are needed for optimal efficacy. Variant surveillance also helps adapt testing strategies, contact tracing, and social distancing measures to address variant-specific challenges. Additionally, it enhances global surveillance and enables timely containment of outbreaks through international collaboration.

## Detection and surveillance measures

China has implemented enhanced surveillance measures to monitor and detect the XBB variants, and preliminary approval has been granted for two novel vaccines targeting XBB omicron subvariants^5^. These vaccines aim to protect high-risk groups, including the aged and people with compromised immune systems. By prioritising the protection of these vulnerable populations, China seeks to manage COVID-19 infections and minimise symptom severity proactively.

Despite the recent increase in cases, health officials have reassured the public that reinfections generally result in milder symptoms and hospitals are not as overwhelmed as they were during the previous winter. Although strict restrictions similar to the zero-COVID era have not been reinstated, guidance has been provided for the elderly and immunosuppressed individuals to avoid crowded areas and mask usage is encouraged in many hospitals. Daily activities have resumed for most Chinese citizens with minimal disruption, and the impact of the new wave of cases has remained relatively limited. The future reinstatement of the zero COVID policy in China remains uncertain.

Genomic sequencing has played a vital role in studying and monitoring severe acute respiratory syndrome coronavirus 2 (SARS-CoV-2) variants in China. A study analysed genomic data from domestic cases between September 2022 and January 2023, revealing specific lineages’ prevalence and temporal dynamics^4^. Notable lineages included BA.5.2.48, BF.7.14, and BA.5.2.49. Genomic surveillance of imported cases also provided information on the proportion of imported cases associated with various lineages. The study highlighted a higher prevalence among men and specific age ranges. The number of cases increased during the study period, peaking in early January 2023.

China has implemented various tracking and surveillance techniques, such as drones, CCTV cameras, digital barcodes, and mobile apps, to combat the COVID-19 outbreak. While these measures have successfully limited the virus’s spread, concerns about data security and privacy have been raised. However, such concerns have not been prominent among the local population.

Early detection plays a crucial role in controlling the transmission of infectious diseases, including anticipated variants such as XBB 1.16 in China^6^. It allows for prompt implementation of isolation, contact tracing, quarantine, targeted interventions, vaccination campaigns, and public awareness initiatives. Early detection also facilitates effective monitoring and surveillance of variant spread, providing essential data on transmissibility, severity, and impact in vulnerable populations. International collaboration and data sharing remains vital for enhancing early detection efforts and fostering coordinated responses to emerging variants.

## International collaboration and lessons learned

The global response to the emergence of new variants of the SARS-CoV-2 virus requires intensified international collaboration and the sharing of valuable insights. Recognising the challenges these variants pose, countries worldwide increasingly acknowledge the imperative to unite their efforts to combat these threats effectively. At the heart of the international collaboration lies the exchange of genomic sequencing data, enabling the rapid identification and tracking of new variants. By sharing information on the genetic composition of these variants, countries can quickly detect and respond to their spread. This timely detection facilitates the implementation of targeted measures to contain the variants and curb their further transmission.

International collaboration also plays a pivotal role in comprehending the characteristics of the new variants. Through exchanging scientific knowledge and research findings, countries can acquire vital insights into these variants’ transmissibility, severity, and potential impact on public health. Such insights prove instrumental in developing effective strategies to control the spread of these variants and mitigate their impact on populations. Furthermore, collaboration in surveillance and monitoring efforts is of critical importance. Countries can exchange best practices and methodologies for monitoring the circulation and prevalence of the new variants within their respective populations. This involves implementing robust testing strategies, contact tracing mechanisms, and isolation measures to identify and contain cases associated with variants. Sharing lessons learnt in these domains enables countries to optimise their surveillance systems and respond more effectively to the challenges posed by the variants.

Ensuring equitable access to vaccines and therapeutics is another vital facet of international collaboration. With the emergence of new variants, it becomes imperative to guarantee the continued effectiveness of vaccines and treatments against these variants. Through collaboration, countries can exchange data on the efficacy of existing vaccines and devise strategies to address potential gaps. In addition, collaboration facilitates the rapid development, evaluation, and distribution of updated vaccines or booster doses tailored to the new variants.

International collaboration also allows the pooling of resources and expertise in research and development. Working collectively, countries can conduct studies to evaluate the efficacy of different interventions against the new variants, encompassing vaccines, therapeutics, and nonpharmaceutical measures. This collaborative research effort enhances the collective understanding of the new variants and informs evidence-based decision-making regarding public health measures.

## Future directions and recommendations

In order to effectively address the challenges presented by the emergence of new variants of the SARS-CoV-2 virus, it is imperative to identify future directions and provide recommendations for global efforts. These directions and recommendations aim to bolster international collaboration and response strategies to navigate the evolving landscape of the pandemic effectively.

Looking ahead, several key areas warrant attention. The first among them is the imperative to strengthen genomic surveillance capabilities. This necessitates prioritising investments in advanced sequencing technologies, establishing robust systems for real-time data collection and analysis, and implementing standardised protocols for the timely sharing of genomic data. Enhancing our ability to monitor new variants’ genomic evolution and transmission patterns enables us to detect better and track their spread, enabling proactive public health responses. Another pivotal aspect is facilitating international data sharing. Encouraging and facilitating the exchange of genomic sequencing data, research findings, and epidemiological information among countries will yield a more comprehensive understanding of the dynamics of new variants. This exchange of information will accelerate the dissemination of knowledge, foster collaboration, and ultimately contribute to a global understanding of the challenges at hand.

Furthermore, it is paramount to continue research efforts investigating emerging variants’ characteristics. This includes studying their transmissibility, severity, impact on vaccine efficacy, and potential for immune escape. Prioritising multidisciplinary collaborations and large-scale studies will generate robust evidence that informs decision-making and enables effective public health responses.

Vigilance in vaccine development and adaptation is also crucial. Ongoing monitoring of the effectiveness of existing vaccines against new variants is vital, and research efforts should concentrate on developing updated vaccines or booster doses tailored specifically to target emerging variants of concern. Collaboration among vaccine manufacturers, regulatory bodies, and public health agencies is essential to accelerate these interventions’ development, evaluation, and deployment. In addition, along with these scientific endeavours, fortifying public health measures remains paramount. Governments must continue emphasising and enforcing preventive measures such as mask use, hand hygiene, physical distancing, and improved ventilation in indoor spaces. Concurrently, effective testing, contact tracing, and isolation protocols are indispensable tools for preventing and controlling the transmission of new variants.

Looking to the future, several recommendations can guide global efforts in addressing the challenges posed by new variants. First, international coordination is of utmost importance. Governments, international organisations, and public health agencies should establish regular communication channels and coordination mechanisms to share information, best practices, and lessons learned rapidly. Collaborative platforms should be established to enable timely exchanges and joint decision-making on global strategies.

Furthermore, risk communication is of critical significance. Transparent and timely communication of the risks associated with new variants is necessary to cultivate public trust and ensure compliance with preventive measures. Governments and health authorities should actively engage with the public, providing accurate information, addressing concerns, and promoting evidence-based practices to mitigate the impact of new variants. In addition, there exists an urgent need for multilateral support for low-income and middle-income countries. International organisations and wealthier nations should increase financial and technical assistance to strengthen these regions’ surveillance, detection, and response capacities. This support will ensure that countries with limited resources can effectively detect, track, and respond to new variants, thereby minimising the global impact of the pandemic.

## Conclusion

China has implemented comprehensive measures to manage COVID-19 cases linked to XBB variants, including enhanced surveillance and targeted strategies for vulnerable groups. International collaboration and data sharing are crucial to tackling these challenges. Strengthening genomic surveillance and standardised data sharing, along with research on variant characteristics and vaccine efficacy, is essential. Vigilance in vaccine development and adaptation is vital, including monitoring effectiveness and developing tailored vaccines or boosters. Reinforcing public health measures and effective risk communication is critical. International coordination and support for low-income and middle-income countries are recommended. Regular communication channels and increased assistance facilitate information sharing and a coordinated response. Following these recommendations, the global community can better manage COVID-19 cases associated with XBB variants in China.

## Ethical approval

NA.

## Consent

NA.

## Sources of funding

No external funding was received for this study.

## Author contribution

G.O. and N.A.: conceptualization. All authors contributed in writing.

## Conflicts of interest disclosure

The authors declare that they have no financial conflict of interest with regard to the content of this report.

## Research registration unique identifying number (UIN)

1. Name of the registry: Not applicable.
2. Unique Identifying number or registration ID: Not applicable.
3. Hyperlink to your specific registration (must be publicly accessible and will be checked): Not applicable.

## Guarantor

Nicholas Aderinto.

## Data availability statement

Data sharing is not applicable to this article.

## Provenance and Peer Review

Not commissioned, externally peer-reviewed.
